# Marsupials and Multi-Omics: Establishing New Comparative Models of Neural Crest Patterning and Craniofacial Development

**DOI:** 10.3389/fcell.2022.941168

**Published:** 2022-06-23

**Authors:** Axel H. Newton

**Affiliations:** The School of BioSciences, University of Melbourne, Parkville, VIC, Australia

**Keywords:** NCC, mammal, heterochrony, constraint, evolution, GRN, skull

## Abstract

Studies across vertebrates have revealed significant insights into the processes that drive craniofacial morphogenesis, yet we still know little about how distinct facial morphologies are patterned during development. Studies largely point to evolution in GRNs of cranial progenitor cell types such as neural crest cells, as the major driver underlying adaptive cranial shapes. However, this hypothesis requires further validation, particularly within suitable models amenable to manipulation. By utilizing comparative models between related species, we can begin to disentangle complex developmental systems and identify the origin of species-specific patterning. Mammals present excellent evolutionary examples to scrutinize how these differences arise, as sister clades of eutherians and marsupials possess suitable divergence times, conserved cranial anatomies, modular evolutionary patterns, and distinct developmental heterochrony in their NCC behaviours and craniofacial patterning. In this review, I lend perspectives into the current state of mammalian craniofacial biology and discuss the importance of establishing a new marsupial model, the fat-tailed dunnart, for comparative research. Through detailed comparisons with the mouse, we can begin to decipher mammalian conserved, and species-specific processes and their contribution to craniofacial patterning and shape disparity. Recent advances in single-cell multi-omics allow high-resolution investigations into the cellular and molecular basis of key developmental processes. As such, I discuss how comparative evolutionary application of these tools can provide detailed insights into complex cellular behaviours and expression dynamics underlying adaptive craniofacial evolution. Though in its infancy, the field of “comparative evo-devo-omics” presents unparalleled opportunities to precisely uncover how phenotypic differences arise during development.

## 1 Introduction

One of the most remarkable, yet enigmatic aspects of the vertebrate skull is the broad diversity of craniofacial shapes observed between species. While our understanding of craniofacial biology has been significantly enhanced through investigations across several vertebrate models, we still know very little about the processes that drive the development of distinct craniofacial adaptations. Comparative embryology and developmental biology in jawed and jawless vertebrates have revealed that craniofacial morphogenesis is driven by a transient population of embryonic progenitors called the neural crest (Kuratani et al., 2018; Fish, 2019). Multipotent neural crest cells (NCCs) direct patterning and development of the head and neck, amongst other structures, and are controlled by deeply conserved gene regulatory networks (GRNs) constituting a species-generic program (Green et al., 2015; Martik and Bronner, 2021). The combination of these developmental and evolutionary observations, with forward genetics and human clinical models of craniofacial disease, have provided a holistic understanding of how the craniofacial prominences are patterned and skull bones develop (Wilkie and Morriss-Kay, 2001; Szabo-rogers et al., 2010; Murillo-Rincón and Kaucka, 2020). However, despite this fundamental understanding of craniofacial biology across vertebrates, we still know remarkably little about how species-specific diversity arises and is patterned during development.

One way we can begin to address this phenomenon is by utilizing comparative models to quantitatively examine how disparities or similarities arise during development. These models need to be suitably chosen depending on the hypothesis being tested. i.e., examining closely related species with unique skull morphologies (disparity), versus distantly related species with similar skull morphologies (convergence). Mammals provide excellent examples to address these hypotheses, owing to their conserved anatomy yet remarkable craniofacial disparity or convergence, shared developmental patterns, heterochrony and lineage-specific constraints, and appropriate divergence times, e.g., within orders or across clades. Through application of these models, we can begin to tease apart how facial morphogenesis and shape diversity is regulated at the cellular and molecular level (Newton et al., 2017; Usui and Tokita, 2018; Newton and Pask, 2020), informing new models of development.

In this article, I outline my perspectives on establishing new comparative mammalian models for investigations into the developmental basis of craniofacial patterning. I discuss the underlying biology of craniofacial morphogenesis, including NCC biology, its influence on patterning, and heterochrony between therian mammals. I emphasize the importance of establishing an appropriate marsupial model for comparative investigations with the eutherian laboratory mouse, including the establishment and utilization of transgenic approaches. Finally, I discuss how single-cell multi-omic approaches, regularly utilized in developmental biology, should be applied to comparative craniofacial models to scrutinize differential cell and molecular behaviours underlying mammalian craniofacial patterning and shape diversity. Establishing a marsupial model for comparative mammalian biology will strengthen our understanding of craniofacial development and how morphological diversity is generated throughout evolution.

### 2 Neural Crest Cells and Patterning of the Head

**FIGURE 1:**
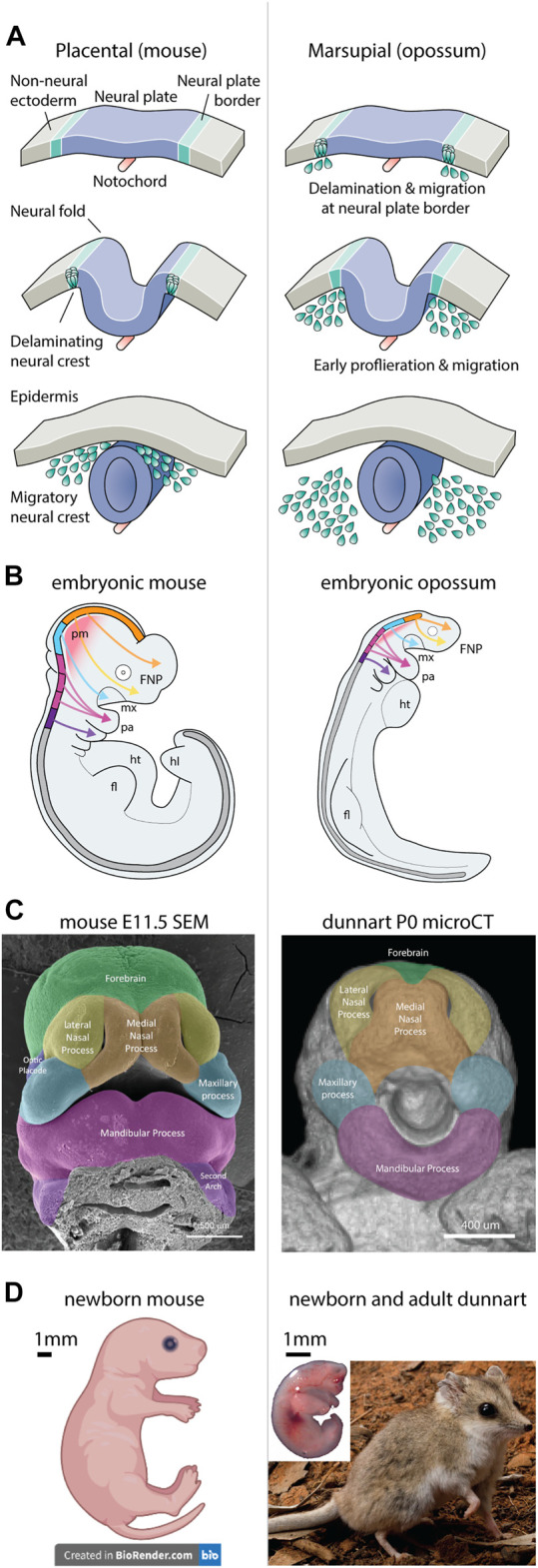
Neural crest and craniofacial development between therian mammals. Craniofacial heterochrony between therian mammals arises from altered neural crest cell behaviours. **(A)** In placental mammals, the neural crest forms in the neural folds and delaminates from the neural tube to migrate throughout the embryo. In marsupials however, the neural crest forms and delaminates from the neural plate border, leading to accelerated migration in the early embryo—redrawn from (Martik and Bronner, 2021). **(B)** Neural crest migration pathways are shared between therian mammals, though are accelerated in marsupials relative to developmental stage. Note marsupials display rapid development of the facial complex and forelimbs, while the CNS and hindlimbs are rudimentary. **(C)** The facial prominences of newborn marsupials resemble those observed in an embryonic mouse (credit FaceBase.org) (Samuels, B. D., 2020). **(D)** Comparative images of newborn mouse and dunnart, demonstrating the altriciality of the dunnart at birth. The adult dunnart superficially resembles a mouse. Image credits: dunnart newborn—Laura Cook; Dunnart Adult—David Paul—Museums Victoria; mouse pup—created with BioRender.com. Abbreviations: fl, forelimb; FNP, frontonasal process; hl, hindlimb; ht, heart; md, mandibular process; mx, maxillary process; pa, pharyngeal arch; pm, paraxial (head) mesoderm.

Development of the vertebrate head and craniofacial skeleton is achieved largely through the contribution of migratory NCCs. NCC specification is regulated through a deeply-conserved GRN comprised of shared suites of core transcriptional regulators, constituting a species-generic program (Green et al., 2015; Martik and Bronner, 2021). During early embryogenesis, NCCs arise within the neuroectoderm at the neural plate border (Figure 1A). Initially, WNT, FGF, and BMP signalling pathways define the border and initiate pre-migratory NCC specification in response to activation of *SOX9* (Cheung and Briscoe, 2003). Committed NCCs undergo activation of epithelial-to-mesenchymal transcription factors, *SOX10, SNAIL* and *SLUG*, and other NCC-specific transcription factors such as *MSX1* and *TFAP2A* (Martik and Bronner, 2021), causing the cells to delaminate and migrate away from the forming neural tube (Figure 1A). The spatial location of NCCs along the anterior-posterior axis of the embryo predefine their paths of migration. The anterior-most cranial NCCs of the forebrain and hindbrain populate the frontonasal process and maxillary arch (Figures 1B,C), contributing to development of the facial skeleton, whereas more posterior cranial NCCs populate the pharyngeal arches to form the musculoskeletal elements of the lower jaw and neck (Figures 1B,C). NCC migration into their target primordia occur in response to cues within the local extracellular environment. Here, as NCCs populate the developing prominences, reciprocal FGF, BMP, SHH, and retinoic acid signalling interactions between mesenchymal NCCs and the epithelial ectoderm and endoderm direct their spatial organization and activate GRNs responsible for proliferation, outgrowth and differentiation of the craniofacial skeleton [(Creuzet et al., 2004; Minoux and Rijli, 2010; Dash and Trainor, 2020; Murillo-Rincón and Kaucka, 2020) and references within].

#### 2.1 The Origin of Species-Specific Pattern

The specific influence of NCCs patterning the vertebrate head has been showcased through cross-species transplantations and xenografts. These experiments have revealed that NCCs possess intrinsic programming and autonomous behaviours which drive species-specific patterning (Schneider, 2018). NCC transplantation chimeras in avian embryos see recipient species develop donor-specific patterning, bone formation and craniofacial morphology (Schneider and Helms, 2003; Chen et al., 2012; Hall et al., 2014; Ealba et al., 2015). Such morphological outcomes are driven *via* intrinsic NCC behaviours, including donor-specific regulation of the cell-cycle and distinct expression of transcriptional regulators and signalling factors (Hall et al., 2014). These unique NCC behaviours are further suggested to influence their local environment to produce distinct morphological outcomes. Here, NCC-derived signals modulate activation of reciprocal signalling pathways to the surrounding ectoderm and endoderm which determine gene expression and spatiotemporal patterning of the facial primordia (Schneider, 2018). In agreement with this, while shared (species-generic) genes and patterning factors are active in the developing facial prominences, each species displays distinct expression profiles during beak outgrowth and development (Wu et al., 2004; Wu et al., 2006; Brugmann et al., 2010). Together, these data suggest that intrinsic species-specific NCC programming influences interactions with their local environment, regulating differentiation of craniofacial cells and tissues and the development of distinct morphological identities.

#### 2.2 Marsupial Heterochrony, Accelerated Neural Crest Cell Specification and Migration

The mechanisms underlying mammalian neural crest patterning and craniofacial development have been largely ascertained from studies in mouse. However, while these findings may be relevant for eutherian mammals, comparative studies in marsupials have revealed pronounced heterochrony in their NCC behaviours. During specification at the neural plate border, marsupial NCCs undergo rapid delamination and migration prior to neural plate folding (Figure 1A) (Smith, 1997; Smith, 2001; Smith, 2020; Vaglia and Smith, 2003; Wakamatsu et al., 2014), leading to large accumulations of NCCs within the forming facial prominences at an earlier equivalent developmental stage to that seen in eutherians (Figure 1B) (Smith, 2001). Remarkably, very little is known about the molecular regulation of marsupial NCCs during development. Two related studies revealed that in the opossum embryo, NCC specification and delamination is accelerated as a result of sequence alteration in a *SOX9* enhancer which drives early activation of *SOX9* in the neural plate border (Wakamatsu et al., 2014; Wakamatsu and Suzuki, 2019). This accelerated activation likely influences the heterochronic migration, proliferation, and ossification observed in marsupials, though no other studies have interrogated these processes or drawn comparisons with eutherians. This represents a large gap in our understanding of how NCC behaviours influence development and differ between distinct mammalian clades. Future studies should address the genetic underpinning of these behavioural differences and their contribution to craniofacial patterning. Curiously, it remains to be seen whether transplantation of marsupial NCCs into recipient mouse embryos (or vice versa) would retain their heterochronic behaviours and promote differential establishment of the facial prominences and skeleton. As such, observations into marsupial NCC biology and comparative heterochrony between eutherians are required for a complete understanding of mammalian NCC patterning and craniofacial development.

### 3 Craniofacial Patterning, Disparity, and Convergence in Therian Mammals

Mammals have evolved unique cranial adaptations which distinguish them from other vertebrates. Evolutionary novelties such as a hinged jaw, middle ear bones and muzzle or semi-motile snout (Higashiyama et al., 2021) have allowed mammals to adapt to a diverse range of ecological niches. However, since diverging ∼160 million years ago (Bininda-Emonds et al., 2007), therian mammals (marsupials and eutherians) have evolved distinct reproductive and developmental strategies, resulting in heterochrony and lineage-specific constraints (Smith, 1997). The marsupial mode of reproduction requires well-developed jaws and forelimbs at a comparatively early stage to allow the altricial neonate to crawl from the birth canal into pouch and attach to the teat for an extended period of suckling. These distinct functional requirements require differences in the onset of development of the olfactory and central nervous system, and musculoskeletal element of the head body and limbs (Smith, 1997; Nunn and Smith, 1998; Weisbecker et al., 2008; Sears, 2009; Keyte and Smith, 2010; Keyte and Smith, 2012; Chew et al., 2014). Particularly, during craniofacial development, ossification and suture closure of the facial bones are advanced to meet the functional requirements associated with suckling (Sánchez-Villagra et al., 2008; Rager et al., 2014; Spiekman and Werneburg, 2017; Cook et al., 2021). Overall, these constraints imposed on the marsupial orofacial bones are suggested to limit evolvability of their cranial anatomy.

Marsupials have evolved altered patterns of cranial modularity (Goswami, 2006; Goswami, 2007; Goswami et al., 2009; Goswami et al., 2012; Goswami et al., 2016) which are thought to reduce their overall skull shape diversity compared to eutherians (Bennett and Goswami, 2013; Fabre et al., 2021). For example, the marsupial jaws form a functionally constrained module, while the frontonasal bones and neurocranium are under relaxed constraint and can evolve more freely (Goswami et al., 2016). On the other hand, eutherian mammals largely lack these constraints during development, thus their cranial bone groups are free to evolve independently producing a greater range of morphological adaptations. Importantly, these frontonasal, jaw and neurocranium bone groups (anatomical modules) possess distinct embryonic origins, arising from the cranial NC, first arch NC, or head mesoderm, respectively (developmental modules) (Couly et al., 1993; Jiang et al., 2002; Yoshida et al., 2008). These semi-independent origins, known as mosaicism, allow flexibility in how different cranial morphologies can evolve and change (Felice and Goswami, 2018), even in the presence of functional constraints. Importantly, the combination of cranial mosaicism with cell-autonomous programming of NCCs provide clues as to how particular cranial adaptations can arise during evolution. Specifically, it can be hypothesized that evolution within GRNs associated with cranial progenitor cell types can produce adaptive morphological outcomes.

These evolutionary hypotheses have been recently applied, investigating the origins of the remarkable craniofacial convergence observed between the marsupial thylacine and eutherian wolf (Goswami et al., 2011; Feigin et al., 2018). During postnatal ontogeny, the thylacine and wolf frontonasal and neurocranial bones develop with strong shape convergence, whilst the thylacine’s maxillary bones (upper jaw) possess constrained shape shared with other marsupials and disparate patterns to that seen in the wolf (Newton et al., 2021). This supports the notion that adaptive evolution (similarity and disparity) of the mammalian skull is modular (Goswami, 2006; Goswami and Polly, 2010), facilitated by mosaic evolution of select bone groups. Furthermore, the distinct embryological origins of the convergent bone groups observed between the thylacine and wolf suggest their underlying GRNs may be convergently targeted by selection. Indeed, comparative genomic investigations of the loci underlying the thylacine and wolf’s cranial convergence revealed enrichment of homoplasy in GRNs associated with cranial mesenchyme migration, differentiation, and ossification (Feigin et al., 2019). Taken together, these studies support the hypothesis that evolution within GRNs of embryonic cranial precursors may specify species-specific patterning of the facial primordia, ultimately influencing craniofacial shape. However, this hypothesis requires further validation, particularly into the role of mammalian NCC heterochrony during early facial development and patterning. As such, establishing a marsupial model of NCC patterning and craniofacial biology that is amenable to manipulation is essential to our understanding of how evolutionary adaptations are produced during development.

### 4 A Marsupial Model to Investigate Mammalian Heterochrony

Modern studies of NCC development and craniofacial patterning in mammals have leveraged the mouse Cre-Lox system, with several transgenic reporter lines established to target various stages of the NCC or skull developmental pathway (Zhang et al., 2002; Rodda and McMahon, 2006; Yoshida et al., 2008; Stine et al., 2009; Rauch et al., 2010; Lewis et al., 2013). Of these, the *Wnt1-cre* strain has been widely utilized for NCC developmental biology to uncover the spatiotemporal decisions underlying NCC differentiation (Soldatov et al., 2019), defining tissue boundaries between NCC and non NCC-derived cranial structures (Jiang et al., 2002; Hu et al., 2004; Lewis et al., 2013), as well as a multipotent NCC line to define models of differentiation (Ishii et al., 2012; Nguyen et al., 2018). In addition, chromatin profiling of mouse NCCs and craniofacial prominences have annotated the regulatory landscape of craniofacial enhancers and putative GRNs (Visel et al., 2009a; Visel et al., 2009b; Attanasio et al., 2013; Pennacchio et al., 2018). Yet while these tools provide powerful and valuable outcomes, they are scarcely utilized outside murine models, limiting comparative mammalian research. Recently however, several new marsupial resources are actively being established, including a pioneering study to generate the first genetically modified marsupials—founder lines of tyrosinase knockout opossums (Kiyonari et al., 2021). Though marsupial transgenic resources are still in their infancy, these advances have primed the generation of new marsupial Cre-Lox resources for developmental investigations. Of note, the generation of a marsupial orthologous *WNT1-cre* line would allow targeted labelling of neural crest cells and their craniofacial derivatives, opening the door for comparative developmental studies and investigations into mammalian heterochrony.

#### 4.1 The Dunnart as the Gold-Standard Marsupial Model

In the past, several marsupial species have provided insights to various aspects of mammalian biology, reproduction, and development (Selwood and Coulson, 2006), with the American opossum (*Monodelphis domestica*) informing models of NCC and limb development (Martin and Mackay, 2003; Vaglia and Smith, 2003; Goswami et al., 2012; Beiriger and Sears, 2014). However, *Monodelphis* are basal American marsupials (superorder Ameridelphia) possessing an 80-million-year divergence from Australian marsupials, similar to times shared between human and mouse. Therefore, an Australian laboratory-based marsupial model with similar easy husbandry, year-round breeding and experimental manipulation is still required for a more complete understanding of mammalian (and marsupial) biology. Dunnarts (*Sminthopsis* sp.; superorder Australidelphia) are small, carnivorous, mouse-like marsupials that are easy to maintain, possess simple husbandry and are polyovular, poly-oestrous and spontaneous ovulators which produce multiple litters of up to 10 pouch young year round (Frigo and Woolley, 1996; Frigo and Woolley, 1997; Suárez et al., 2017; Cook et al., 2021). Owing to this, several new resources are being established for the fat-tailed dunnart (*S. crassicaudata*, hereafter referred to as the dunnart) as the gold-standard model for next-gen marsupial biology. These include a chromosome level assembly, transcriptomic and gene regulatory datasets, induced pluripotent cells, inbred strains, and transgenic laboratory lines (Eldridge et al., 2020). Furthermore, like other marsupials, the dunnart possesses significant heterochrony in development of its head, brain and limbs compared with eutherian species, making it an excellent model for comparative mammalian research.

One of the most remarkable features of dunnart biology is its rapid gestation and ultra-altricial state at birth (Suárez et al., 2017; Cook et al., 2021). Dasyurid marsupials, including the dunnart, represent some of the most altricial of all extant mammals. Dunnart neonates are born after a rapid 13.5-day gestation, compared to ∼20 days in mouse (Figure 1D), and superficially resemble a eutherian foetus. At birth, the dunnart orofacial region appears as rudimentary facial prominences akin to an embryonic day 11.5–12 mouse (Figure 1C), despite being functional to accommodate suckling. The newborn dunnart lacks a developed brain and has paddle-like hindlimbs, but possesses highly developed, muscularized forelimbs with claws to accommodate crawling (Figure 1D) (Suárez et al., 2017; Cook et al., 2021). Remarkably, newborn dunnarts lack mineralized bone in the facial skeleton and forelimbs, which rapidly ossify within the first 24 h, while the hindlimbs do not start to ossify until ∼D5 (Cook et al., 2021). This extreme heterochrony and altriciality at birth allows direct manipulations of these developmental systems *ex utero*, at equivalent eutherian embryonic stages (Paolino et al., 2018). Critically, the ultra-altricial birth of dasyurids demand additional acceleration of the onset of NCC specification, migration and proliferation, compared to the opossum (Smith, 2020). These features distinguish the dunnart as an exceptional mammalian model to investigate NCC-derived craniofacial patterning and ossification. However, detailed analyses which substantiate these early NCC behaviours in *Sminthopsis* have yet to be performed, representing an important first step to understand their NCC biology and thus heterochrony in mammals*.* Nevertheless, the dunnart is well positioned to determine how altered developmental timing influences ontogeny and craniofacial morphogenesis, providing new insights into the origin of species-specific pattern.

### 5 A Look to the Future: Comparative Evo-Devo-Omics

The age of comparative and functional genomics has accelerated investigations into the molecular basis of mammalian trait evolution. Comparative genomics has allowed identification of genes and regulatory regions under selection within and between lineages (Capra et al., 2013; Parker et al., 2013; Foote et al., 2015; Feigin et al., 2018, 2019); comparative bulk RNA-seq has revealed differentially expressed genes between tissues or developing structures (Eckalbar et al., 2016; Cooper et al., 2020); and chromatin pulldown or accessibility assays (ChIP, HiC, or ATAC-seq) define the gene regulatory landscape associated with these tissues or developing structures (Visel et al., 2009a; Attanasio et al., 2013). However, though powerful, individually these analyses are static and may overlook dynamic processes that contribute to development of complex traits. For example, while identification of differentially expressed genes or enhancers active in the embryonic orofacial region may constitute components that contribute to mammalian facial shape diversity (species-generic), such analyses are unable to capture dynamic regulation of these and the GRNs that influence development of unique anatomical features (species-specific) (Schneider, 2018)—as exemplified in avian models (Schneider and Helms, 2003; Chen et al., 2012; Hall et al., 2014; Ealba et al., 2015). As such, alternative approaches are required to disentangle the complex landscape of craniofacial development between disparate species.

The advent of single-cell omics has revolutionized developmental biology, producing high-resolution atlases of diverse developmental processes (Cao et al., 2019). To date, single-cell studies have been applied to multiple aspects of neural crest patterning and craniofacial development (Li et al., 2019; Soldatov et al., 2019; Farmer et al., 2021; Morrison et al., 2021; Pagella et al., 2021; Tatarakis et al., 2021), providing unique insights into how these complex developmental processes are regulated. Single-cell transcriptomics (scRNA-seq) allow detailed characterization of transcriptional profiles and cell-types present within developing structures, fate decisions and gene expression dynamics underlying differentiation of progenitors into mature cell types (Trapnell et al., 2014), and cell-cell signalling interactions between adjacent tissues (Jin et al., 2021). Importantly, scRNA-seq data can be integrated with genome-wide assays for Transposase-Accessible Chromatin (ATAC-seq) to define regulatory elements active within their underlying cell types (Buenrostro et al., 2015; Cao et al., 2018; Stuart et al., 2019), or spatial transcriptomics to resolve cellular gene expression profiles in individual cells (Xia et al., 2019) or developing tissues *in situ* (Marx, 2021). Used in combination, these techniques provide powerful methods to integrate developmental biology with gene expression dynamics and construction of species-specific GRNs.

Despite their potential, single-cell multi-omic approaches have been scarcely applied in comparative evolutionary biology. Such studies of “comparative evo-devo-omics” between taxa are becoming rapidly viable to investigate the molecular mechanisms underlying convergence, constraint, or innovation in specific developmental processes (Shafer, 2019; Mahadevaiah et al., 2020). However, the lack of these applied studies are largely in response to significant technical limitations surrounding integration and batch correction of disparate datasets, specificity and stage-matching of tissues and homologous cell types between disparate species, and quality of the underlying genome and transcriptome—reviewed by (Shafer, 2019). Nevertheless, these limitations can be mitigated through application of tools aiding dataset integration, sequencing depth and batch correction, as well as issues with transcriptome quality and gene orthology (Butler et al., 2018; Haghverdi et al., 2018). Furthermore, new applied methodologies are being produced to better identify and match homologous cell and tissue types (Tosches et al., 2018; Welch et al., 2019; Feregrino and Tschopp, 2021) and their proportions between distantly related species and datasets (Phipson et al., 2021). Given the rapid rate by which these limitations are being resolved by the community, comparative evo-devo-omics presents a powerful platform to interrogate the cell, molecular and developmental mechanisms underlying heterochronic NCC specification and facial patterning between marsupial and eutherian mammals.

#### 5.1 Mammalian Craniofacial Heterochrony at Single Cell Resolution

**FIGURE 2:**
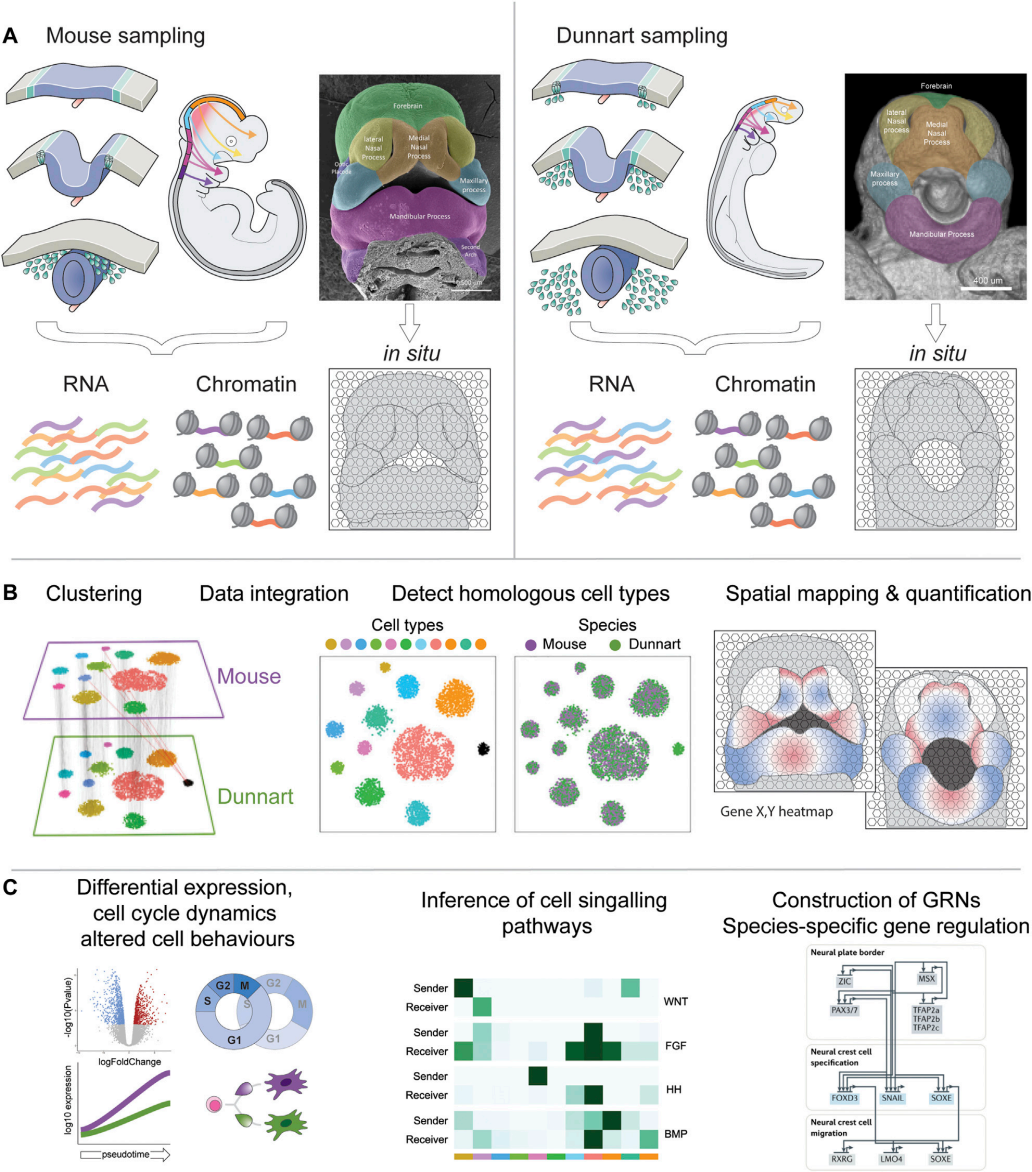
Workflow for comparative craniofacial single-cell multiomics. **(A)** Single cells can be isolated from dunnart and mouse NCCs and developing craniofacial prominences for multiplexed isolation and sequencing of RNA and open chromatin. Facial tissue sections can be processed for *in situ* spatial transcriptomics or MERFISH (Xia et al., 2019). Single-cell RNA and ATAC-seq data can be readily integrated using pipelines such as Seurat (Butler et al., 2018). **(B)** Dunnart and mouse datasets can be individually clustered or integrated to generate an atlas of homologous cell type populations—adapted from (Stuart et al., 2019). Cell transcriptomic and epigenetic profiles can be further mapped back to their spatial organization in the embryo. **(C)** The combination of these methods allows sophisticated downstream workflows to examine differential expression between species-specific clusters (pseudobulk) or cell lineage differentiation (pseudotime), inference of cell-cell signalling relationships (Jin et al., 2021), or construction of GRNs and species-specific patterns of gene regulation (Martik and Bronner, 2021).

Using the above examples, I present a hypothetical workflow for detailed investigations into mammalian craniofacial heterochrony and evolution through a comparative lens. First, sampling of single-cell RNA, chromatin and spatial profiles of stage-matched dunnart and mouse embryos (Figure 2A) will allow generation of species-specific transcriptional atlases, building on existing datasets (Soldatov et al., 2019) and producing novel mammalian resources. The resulting transcriptomic, epigenetic and spatial profiles can be clustered and integrated for detection of homologous and novel cell types (Tosches et al., 2018; Welch et al., 2019), conserved and disparate gene co-expression modules (Feregrino and Tschopp, 2021), cell type proportions (Phipson et al., 2021) and spatial quantification of genes *in situ* (Figure 2B). From here, evolutionary hypotheses can be tested through identification of dynamic transcriptional, signalling, epigenetic and spatial relationships, revealing shared (species-generic) and unique (species-specific) processes underlying facial development and evolution (Figure 2C). Through application of these approaches, we can begin to determine how NCC gene regulatory architecture differs between therian mammals, and their influence on heterochronic craniofacial patterning. Ultimately, this will not only provide valuable data into how diverse facial shapes are produced during development, but also provide novel insights into how mammalian craniofacial diversity arises during evolution.

## Data Availability

The original contributions presented in the study are included in the article/Supplementary Material, further inquiries can be directed to the corresponding author.
